# High burden of malaria infection in pregnant women in a rural district of Zambia: a cross-sectional study

**DOI:** 10.1186/s12936-015-0866-1

**Published:** 2015-09-30

**Authors:** Enesia Banda Chaponda, Daniel Chandramohan, Charles Michelo, Sungano Mharakurwa, James Chipeta, R. Matthew Chico

**Affiliations:** Department of Biological Sciences, University of Zambia, Lusaka, Zambia; Department of Disease Control, Faculty of Infectious and Tropical Diseases, London School of Hygiene and Tropical Medicine, London, UK; Department of Public Health, School of Medicine, University of Zambia, Lusaka, Zambia; Africa University, Mutare, Zimbabwe; Johns Hopkins Bloomberg School of Public Health, Baltimore, USA; Department of Paediatrics and Child Health, University of Zambia School of Medicine, P.O. Box 50110, Lusaka, Zambia

**Keywords:** Malaria, Pregnancy, Zambia

## Abstract

**Background:**

Malaria continues to be a major health problem in low-income countries. Consequently, malaria control remains a public health priority in endemic countries such as Zambia. Pregnant women and children under 5 years of age are among groups at high risk of malaria infection. Malaria infection is associated with adverse birth outcomes that affect the mother, foetus, and infant. Infection with HIV has been shown to increase the risk of malaria infection in pregnancy. The prevalence and the predictors of malaria infection among pregnant women resident in the Nchelenge District of northern Zambia were investigated.

**Methods:**

Between November 2013 and April 2014, pregnant women in the catchment areas of two health centres were recruited during their first antenatal care visit. HIV testing was conducted as part of routine care. In addition, blood samples were collected from 1086 participants and tested for malaria infection using standard microscopy and polymerase chain reaction (PCR) techniques specific for *Plasmodium falciparum*. Multivariate logistic regression were conducted to examine the predictors of malaria infection.

**Results:**

The prevalence of malaria identified by microscopy was 31.8 % (95 % confidence intervals [CI], 29.0–34.5; N = 1079) and by PCR was 57.8 % (95 % CI, 54.9–60.8; N = 1074). HIV infection was 13.2 % among women on their first antenatal visit; the prevalence of malaria detected by PCR among HIV-uninfected and HIV-infected women was 56.7 % (531/936) and 65.2 % (90/138), respectively. In the final model, the risk of malaria infection was 81 % higher among pregnant women recruited from Nchelenge health centre compared to those attending the Kashikishi health centre (adjusted odds ratio = 1.81; 95 % CI, 1.38–2.37, *P* < 0.001), and HIV-infected women across health centres had a 46 % greater risk of malaria infection compared to HIV-uninfected women (adjusted odds ratio = 1.46; 95 %, 1.00–2.13, *P* = 0.045).

**Conclusion:**

High burden of malaria detected by PCR in these pregnant women suggests that past prevention efforts have had limited effect. To reduce this burden of malaria sustainably, there is clear need to strengthen existing interventions and, possibly, to change approaches so as to improve targeting of groups most affected by malaria.

**Electronic supplementary material:**

The online version of this article (doi:10.1186/s12936-015-0866-1) contains supplementary material, which is available to authorized users.

## Background

Approximately 35 million pregnant women are at risk of malaria infection each year in sub-Saharan Africa [1]. Adverse consequences of malaria infection during pregnancy include maternal anaemia, intra-uterine growth retardation [2], preterm delivery [3], stillbirth [4, 5] and low birth weight [6]. Low birth weight is associated with a marked increase in neonatal death [7–10].

Meta-analysis of malaria in pregnancy (MiP) studies conducted in Eastern and Southern Africa between 1990 and 2011 showed that 32.0 % (95 % confidence intervals [CI], 25.9–38.0; N = 47,433) of pregnant women attending antennal care (ANC) facilities had peripheral parasitaemia. When the time period was restricted to studies conducted between 2000 and 2011, parasitaemia was 29.5 % (95 % CI, 22.4–36.5; n = 18,375) [11]. These estimates were calculated using a standard method for correcting errors of magnitude based on the known specificity and sensitivity of individual diagnostic methods [12].

Various diagnostic methods can be used in malaria detection. In this study microscopy and polymerase chain reaction (PCR) methods were employed. One advantage of standard microscopy is that the method requires a relatively short time for diagnosis when used in areas of high transmission and parasites are present in high concentrations [approximately 1000 parasites per microlitre (µl) of blood]. However, if parasite densities are very low, examination of each slide is labour-intensive [13]. Moreover, the sensitivity and specificity are greatly influenced by the skills and workload of microscopists [13]. In contrast, the use of PCR for the diagnosis of malaria is highly sensitive and consistent in the detection of parasites; PCR has the ability to measure infections where parasite counts are as low as 5 per µl of blood [14, 15]. However, some problems arise with false-negative results when DNA isolation is inappropriate [16, 17].

In Zambia, malaria is endemic in all 10 provinces and *Plasmodium falciparum* is responsible for approximately 95 % of all cases [18]. Since 2006, the National Malaria Control Programme has conducted a Malaria Indicator Survey (MIS) every 2 years to measure the prevalence of malaria by microscopy in children under 5 years of age in selected sites. The prevalence of malaria parasitaemia decreased in Zambia from 2006 to 2010 in some regions, while little change was observed in others [19]. Of concern is that parasitaemia declined between 2006 and 2008 in Eastern, Northern, and Luapula Provinces, but was higher in the 2010 survey. There has been a 48.3 % decline in the national overall malaria prevalence in children under 5 years of age between 2006 and 2012 (22.1 versus 14.9 %), although there was only a 2.5 % reduction in Luapula Province (32.9 versus 32.1 %) [20].

Zambia revised its national malaria drug treatment policy between 2000 and 2005 to adopt artemisinin combination therapy (ACT) as the first-line treatment for uncomplicated malaria [21]. Quinine is the first-line treatment for uncomplicated malaria in pregnancy in the first trimester, whereas ACT is for use in second and third trimesters. For complicated malaria, parenteral quinine is recommended in all trimesters; sulfadoxine-pyrimethamine (SP) is used by policy for intermittent preventive treatment of malaria in pregnancy (IPTp) and also serves as a drug of choice for people who cannot tolerate ACT and during periods of ACT stock-outs in health facilities [22]. The Zambian malaria policy states that IPTp-SP doses are to be administered during pregnancy at scheduled ANC visits, spaced 1 month apart after 16 weeks of gestation. The 2012 MIS reported that 84.6 % of rural and 93.0 % of urban women who had given birth in the five preceding years reported taking at least one dose of IPTp-SP [22].

Infection with HIV has been shown to impair the capacity of pregnant women to control peripheral and placental infection [23]. A review of 11 studies showed that HIV-infected women experience more peripheral and placental malaria, higher parasite densities, and more febrile illnesses, severe anaemia, and adverse birth outcomes than HIV-uninfected women [24].

The prevalence of HIV among ANC attendees in Zambia is estimated to be 16.8 % in urban and 8.3 % in rural areas [25]. In 2010, national HIV testing among ANC attendees was estimated to be 94.0 % [26], and virtually all pregnant women are now tested for HIV. Zambia is listed among the 22 priority African countries with the highest numbers of pregnant women living with HIV who are in need of antiretroviral therapy for the prevention of mother-to-child transmission of HIV [27]. According to the 2013 ‘*Towards Universal Access report on HIV and AIDS’*, Zambia has made notable progress with coverage of maternal antiretroviral treatment (prophylaxis and therapy). The coverage of antiretroviral drugs for prevention of mother-to-child transmission, excluding single dose nevarapine, was estimated to be >95 % (95 % CI, 87—>95 %) [27].

An estimated 200,000 pregnancies in Zambia are at risk of malaria each year [28], however the MIS has not reported any estimates of malaria infection among pregnant women to date. Thus, this study was conducted to determine the prevalence of peripheral parasitaemia and risk factors for malaria infection from a cross-section of first ANC attendees in Luapula Province, an area known to have intense malaria transmission [29]. According to the Zambia Demographic and Health Survey 2013–2014, 94.6 % of women between 15 and 49 years of age in Luapula Province reported having received ANC from a skilled health care provider during pregnancy for their most recent birth in the preceding 5 years [30]. The coverage of IPTp-SP reported in the MIS at provincial level was as follows: 89.7, 76.6, and 57.6 % of women took at least one dose, two or more doses, and three or more doses of IPTp-SP, respectively [20]. The prevalence of HIV among ANC attendees at the two recruitment sites for the study, Kashikishi and Nchelenge health centres, in 2014 was 13.0 and 13.3 %, respectively [31]. This estimate combined new cases and already known HIV positive cases.

## Methods

### Design, population and sampling procedures

This was a prospective cohort study of ANC attendees involving recruitment at ANC booking with follow-up to delivery. The study site was the catchment area for two health centres in Nchelenge District which is located on the shores of lake Mweru and has a population of 173,680 [32]. Recruitment of participants was done at the two health centres between November 2013 and April 2014, the high malaria transmission period.

The main study was designed to estimate the prevalence of malaria, curable sexually transmitted and reproductive tract infections and their co-infection, to explore determinants of poor birth outcomes in pregnancy, and to assess in vivo efficacy of SP over 28 days following administration.

The sample size of 1086 was based on the assumption that the incidence of adverse birth outcomes (low birth weight, stillbirth, preterm delivery or intra-uterine growth retardation) among women who have a malaria infection or a curable sexually-transmitted and reproductive tract infections infection during pregnancy would be at least 10 %. Thus, the sample size had 80 % power with 95 % CIs to detect risk factors that have an odds ratio >2 (n = 984); 10 % (n = 102) more pregnant women were recruited to account for expected losses to follow-up as observed in another MIP study in Zambia [33]. Women were enrolled upon providing informed written consent if they had not been exposed to anti-malarial and/or antibiotic therapy within the previous 4 weeks, were willing to have a member of the study team record their HIV test results following routine HIV screening, and had a gestational age of <32 weeks.

### Data and sample collection

Data and sample collection were conducted alongside the provision of routine ANC activities. Routine HIV testing was done using finger-prick blood with two rapid test kits (Determine^®^ HIV-1/2 [Abbott] and Uni-Gold™ Recombigen^®^ HIV-1/2 [Trinity Biotech]). Women were generally tested together with their spouses/partners. Individuals found to be seropositive using Determine^®^ HIV-1/2 were then tested with Uni-Gold™; HIV status was concluded based on the result of the second test.

Trained field workers administered a questionnaire to participating woman in a private room. The questionnaire was used to collect information on socio-demographics, malaria prevention interventions, HIV status and obstetric history. Finger-prick blood was collected and used to prepare a thick smear from each participant for diagnosis of malaria by microscopy. Approximately 160 μl of peripheral blood was placed on four circles of Whatman^®^ filter paper and labelled with a unique identifying number for each participant and was subsequently used for malaria diagnosis by PCR. IPTp-SP was administered by direct observation to participants whose gestational age was >16 weeks. Women who were receiving cotrimoxazole prophylaxis were not given IPTp-SP due to toxicity concerns. Strips of filter paper containing blood spots were then air-dried and individually stored in envelopes pending deoxyribonucleic acid (DNA) extraction for malaria diagnosis by PCR. Thick blood films were stained using 10 % Giemsa solution and read by two independent microscopists. Parasite density was determined by assuming 8000 white blood cells (WBCs) per µl and counting the number of parasites per 200 WBCs. In cases where fewer than nine parasites were counted against 200 WBCs, parasites were counted against 500 WBCs. Two-hundred high-power fields were read before declaring a slide negative. If results between two microscopists were discordant, a third observer read the slide to determine the diagnosis and parasite count.

DNA extraction was carried out using the Chelex method as described elsewhere [34]. *P. falciparum* was detected using the same nested PCR method described by Snounou et al. [35], with modifications to the PCR parameters [36]. Briefly, all PCR reactions were carried out in total volumes of 25 µl using Thermo Scientific^®^ Dream Taq PCR Master Mix (2X) and 0.5 µM of each primer and 2 µl of template; 2 µl of the primary amplicon was used as a template in the secondary reaction. The primary reaction PCR programme was as follows: initial denaturation at 94 °C for 3 min, followed by 30 cycles of denaturation at 94 °C for 1 min, annealing at 60 °C for 2 min and extension at 72 °C for 2 min. The final extension after the 30 cycles was at 72 °C for 2 min. The secondary PCR reaction consisted of 30 cycles of reaction as above and the annealing temperature was at 55 °C. Negative controls were included in all extraction batches and, separately, a positive and a negative control were added to all PCR runs. The secondary amplicon was analysed by electrophoresis on 2 % ethidium bromide stained agarose gel and visualised under ultra-violet light.

### Data processing and analysis

Data were double-entered in EpiData version 3.1 software [37], cleaned, processed and analysed using Stata software version 13 [38]. Frequencies of all variables were generated to check for missing data. Variables were then recoded. Data on sources of income, level of education, and fixed and durable assets (type of roof and floor, type of fuel used for cooking, ownership of assets such as television, fridges and radios) were used to create an index of household wealth using principal components analysis [39]. Geometric means and medians were used to describe non-normally distributed continuous variables such as parasite density and age. Characteristics of study participants were described using percentages and the Chi-squared test was used to assess differences in proportions between women recruited at the two sites and between HIV-infected and HIV-uninfected women. Standard statistical tests (i.e. Mann–Whitney *U* test and t tests) were used to assess significance (*P* < 0.05) of differences in the distribution of continuous variables. Parasite densities were log-transformed before conducting t-tests or analyses of variance. The prevalence of malaria infection, determined by microscopy or by PCR, and their 95 % CI, were estimated. The pre-specified primary outcome was malaria measured by PCR, therefore, results obtained from PCR diagnosis were used to assess potential risk factors for malaria infection. Univariate analyses of potential predictors of malaria infection were conducted using logistic regression, and crude odds ratios were estimated. Potential predictors that showed significance at *P* < 0.1 in the univariate analysis were entered into a multivariable model and assessed using a likelihood ratio test. Factors that were found to be independently associated with malaria infection at *P* < 0.05 were entered in a final model and adjusted odds ratios obtained. Independent variables in the final model were checked for interaction.

### Ethical considerations

The study protocol was approved by the University of Zambia Biomedical Research Ethics Committee (reference number 004-02-13) and the London School of Hygiene & Tropical Medicine Observational/Interventions Research Ethics Committee (reference number 6292). Participation was voluntary and participants could withdraw at any time. To ensure confidentiality interviews were conducted privately and each participant was assigned a unique identifying number that was used to anonymize biological samples, questionnaires, and result record forms.

Women and/or their partners who tested HIV positive for the first time underwent assessment for eligibility to receive antiretroviral therapy and cotrimoxazole prophylaxis as per routine procedures. All women, except those currently receiving cotrimoxazole prophylaxis, were provided IPTp-SP according to national guidelines. In the rare event that a woman showed up for ANC booking with symptomatic malaria, routine laboratory testing was done using a rapid diagnostic test kit (SD Bioline Malaria Ag P.f/Pan brand; Standard Diagnostics, Inc.) and treatment was administered based on national policy guidelines. Strengthening the reporting of observational studies in epidemiology (STROBE) guidelines (Additional file 1) were followed in reporting this study [40].

## Results

### Participation and distribution

Of the 1237 women who came for ANC booking, 1095 (89 %) were eligible for enrolment. Only 0.8 % (n = 9) of eligible women refused to take part in the study. A total of 142 women were ineligible for the following reasons: six were not pregnant, 116 came after 32 weeks gestation; 13 had taken anti-malarial treatment in the previous 4 weeks; five had taken anti-malarial treatment in the 4 weeks prior to recruitment and were more than 32 weeks pregnant, and two had taken antibacterial agents in the previous 4 weeks. One woman withdrew consent among the 1086 participants who had been successfully recruited.

The median age of participants was 25 years (interquartile range 20–30) and the median gestational age was 22 weeks (interquartile range 19–25.5). Nearly one-half of the participants, 49.2 % (n = 534) reported ownership of a bed net (treated or untreated combined). Of 531 bed net owners, 78.3 % (n = 416) reported having slept under a bed net the previous night. Overall, only 38.5 % (n = 416) of participants slept under a bed net the previous night. Table 1 presents characteristics of women according to the recruitment sites. No association was found between site of recruitment and socio-demographic characteristics of the participants. Socio-demographic details of participants are presented in full within Table 1.

**Table 1:** Baseline characteristics of pregnant women resident in the Nchelenge district of Zambia by recruitment site

Variable	All (N = 1085) n (%) or n, median (IQR)	Kashikishi (n = 747) n (%) or n, median (IQR)	Nchelenge (n = 338) n (%) or n, median (IQR)	*P* value
Age (median)	25 (20–30)	737, 24 (20–30)	338, 25 (20–31)	0.133
Marital status				0.591
Single	203 (18.7)	134 (17.9)	69 (20.4)	
Married	874 (80.6)	607 (81.3)	267 (80.0)	
Widowed/separated	8 (0.7)	6 (0.8)	2 (0.6)	
Years of schooling				0.398
None to 6 years	426 (39.3)	287 (38.4)	139 (41.1)	
7 years and above	659 (60.7)	460 (61.6)	199 (58.9)	
Gravidity				0.657
Primigravidae	261 (24.1)	183 (24.5)	78 (23.1)	
Secundigravidae	165 (15.2)	117 (15.6)	48 (14.2)	
Multigravidae	659 (60.7)	447 (59.8)	212 (62.7)	
Bed net ownership				0.105
No	551 (50.8)	367 (49.1)	184 (54.4)	
Yes	534 (49.2)	380 (50.9)	154 (45.6)	
Bed net usage				0.349
No	666 (61.6)	451 (60.6)	215 (63.6)	
Yes	416 (38.4)	293 (39.4)	123 (36.4)	
Missing^a^	3	0	3	
IRS in previous 1 year				0.851
No	808 (78.1)	563 (75.7)	245 (72.7)	
Yes	226 (21.9)	156 (21.0)	70 (20.8)	
Unknown^a^	51	28	23	
Wealth quintile				0.089
Lowest	217 (20.0)	135 (18.1)	82 (24.3)	
Second	221 (20.4)	151 (20.3)	70 (20.7)	
Middle	214 (19.8)	155 (20.8)	59 (17.5)	
Fourth	215 (19.9)	158 (21.20	57 (16.9)	
Highest	216 (19.9)	146 (19.6)	70 (20.7)	
Missing^a^	2	2	0	
HIV status				0.374
Negative	941 (86.8)	643 (86.2)	298 (88.2)	
Positive	143 (13.2)	103 (13.8)	40 (11.8)	
Missing^a^	1	1	0	
ART among HIV-infected				0.689
No	82 (57.3)	58 (56.3)	24 (60.0)	
Yes	61 (42.7)	45 (43.7)	16 (40.0)	
NA (HIV negative)^a^	941	643	298	

*IQR* interquartile range, *IRS* indoor residual spraying, *ART* antiretroviral therapy, *NA* not applicable

^a^Missing values and NA (HIV Negative) are only presented as numbers and were not included in the calculation of percentages and in the Chi-squared test for association

### Malaria and HIV prevalence

The prevalence of HIV infection was 13.2 % (95 % CI, 11.3–15.3) and the highest burden was observed in multigravidae (Table 2). The prevalence of malaria infection measured by microscopy was 31.8 % (95 % CI, 29.1–34.6) and by PCR was 57.8 % (95 % CI, 54.9–60.8). Parasite density ranged from 64 to 24,760 parasites per µl of blood with a geometric mean of 1082 (95 % CI, 962–1217) asexual parasites per µl of blood. Parasite density was highest among primigravidae with geometric means being significantly different across gravidae (*P* < 0.001). Of the 343 malaria positive samples detected by microscopy, seven were negative for *P. falciparum* by PCR.

**Table 2:** Prevalence of *Plasmodium falciparum* malaria infection and parasite density at first antenatal care visit by gravidity among pregnant women of Nchelenge District in Zambia

Infection and detection method	All % (95 % CI, n/N) or n, mean^a^ (95 % CI)	Primigravidae % (n/N) or n, mean^a^ (95 % CI)	Secundigravidae % (n/N) or n, mean^a^ (95 % CI)	Multigravidae % (n/N) or n, mean^a^ (95 % CI)	*P* value
HIV Rapid tests (Determine^®^ HIV-1/2 and Uni-Gold™)	13.2 (11.3–15.3, 143/1084)	8.0 (21/261)	13.9 (23/165)	15.1 (99/658)	0.018
Malaria PCR	57.8 (54.9–60.8, 621/1074)	68.7 (178/259)	61.8 (102/165)	52.5 (341/650)	<0.001
Malaria Microscopy^b^	31.8 (29.1–34.6, 343/1079)	51.0 (132/259)	37.0 (61/165)	22.9 (150/656)	<0.001
Parasite density (geometric mean)	343, 1082 (962–1217)	132, 1378 (1133–1677)	61, 1164 (897–1509)	150, 848 (714–1010)	<0.001

Of the expected samples from 1085 participants, 1074 were processed by PCR and 1079 by microscopy due to missing samples

*95* *% CI* 95 % confidence interval, *PCR* polymerase chain reaction

^a^Geometric mean

^b^Of the 343 malaria positive samples detected by microscopy seven were found negative by PCR specific for *Plasmodium falciparum* detection

Of the 1074 women whose results were available for malaria as detected by PCR, 8.4 % (n = 90, 95 % CI, 6.7–10.2 %) were co-infected with HIV. Table 3 presents characteristic of HIV-infected and HIV-uninfected women. Infection with HIV was associated with age, gravidity and bed net usage on the night before enrolment. Among women who tested positive for HIV, combined with those with known HIV-positive status, 42.7 % were receiving antiretroviral therapy at the time of recruitment. The prevalence of malaria among HIV-infected and HIV-uninfected women detected by PCR was 65.2 % (90/138) and 56.7 % (531/936), respectively (*P* = 0.059). In the case of microscopy, 31.3 % (294/939) of HIV-uninfected had parasitaemia compared to 35.0 % (49/140) among HIV-infected women (*P* = 0.382). HIV-infected women had significantly higher parasite densities than HIV-uninfected women (*P* < 0.001). Parasite density among HIV-infected women receiving antiretroviral therapy and those who were not at recruitment was not significantly different: 1310 (95 % CI, 693–2476, n = 15) and 2131 (95 % CI, 1408–3224, n = 34), respectively (*P* = 0.189).

**Table 3:** Characteristics of HIV-uninfected and HIV-infected women of Nchelenge district in Zambia

Variable	All (N = 1084) n (%) or n, median (IQR) or n, mean (95 % CI)	HIV-uninfected (n = 941) n (%) or median (IQR) or n, mean (95 % CI)	HIV-infected (n = 143) n (%)or n, median (IQR) or n, mean (95 % CI)	*P* value
Recruitment site				
Kashikishi	746 (68.8)	643 (68.3)	103 (72.0)	0.374
Nchelenge	338 (31.2)	298 (31.7)	40 (28.0)	
Age (median)	25 (20–30)	24 (20–30)	27 (22–32)	<0.001
Gravidity				
Primigravidae	261 (24.1)	240 (25.5)	21 (14.7)	0.018
Secundigravidae	165 (15.2)	142 (15.1)	23 (16.1)	
Multigravidae	658 (60.7)	559 (59.4)	99 (69.2)	
Bed net ownership				
No	550 (50.7)	472 (50.2)	78 (54.6)	0.328
Yes	534 (49.3)	469 (49.8)	65 (45.5)	
Bed net usage on previous night				
No	665 (61.5)	563 (60.0)	102 (71.8)	0.007
Yes	416 (38.5)	376 (40.0)	40 (28.8)	
Missing^a^	3	2	3	
IRS in last 12 months				
No	665 (61.5)	563 (59.9)	102 (71.8)	0.628
Yes	416 (38.5)	376 (40.0)	40 (28.2)	
Unknown	51	46	5	
Wealth index				
Lowest	217 (20.1)	193 (20.6)	24 (16.8)	0.863
Second	220 (20.3)	191 (20.3)	29 (20.3)	
Middle	214 (19.8)	183 (19.5)	31 (21.7)	
Fourth	215 (19.9)	185 (19.7)	30 (20.9)	
Highest	216 (20.0)	187 (19.9)	29 (20.3)	
Missing^a^	2	2	0	
Malaria infection *PCR*				
Negative	453 (42.2)	405 (43.3)	48 (34.8)	0.059
Positive	621 (57.8)	531 (56.7)	90 (65.2)	
Missing^a^	10	5	5	
Microscopy				
Negative	736 (68.2)	645 (68.7)	91 (65.0)	0.382
Positive	343 (31.8)	294 (31.3)	49 (35.0)	
Missing^a^	5	2	3	
Parasite density (geometric mean)	343, 1082 (962–1217)	294, 990 (876–1120)	49, 1836 (1306–2581)	<0.001

*IQR* Interquartile range, *IRS* indoor residual spraying

^a^Missing values are only presented as numbers and were not included in the calculation of percentages and in the Chi-squared test for association

**Fig. 1:**
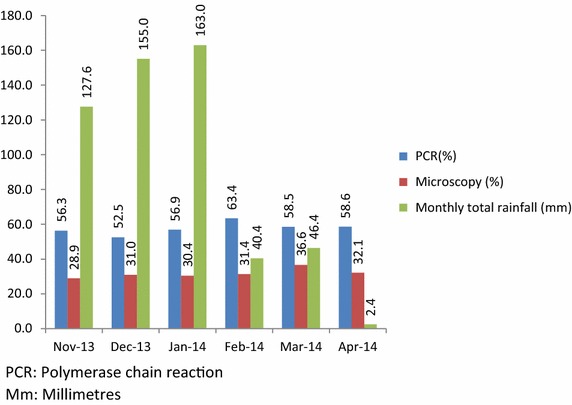
The prevalence of malaria infection and total monthly rainfall over the 6 months study recruitment period

Figure 1 shows the prevalence of malaria infection among study participants each calendar month during the 6-month recruitment period from November 2013 to April 2014. In the univariate analyses of recruitment site, age, marital status, gravidity, bed net ownership, bed net usage, wealth quintiles, and HIV status were associated with malaria infection at *P* < 0.1. In the multivariate analysis of HIV status, site of recruitment and wealth quintile were independently associated with malaria infection (Table 4). Wealth quintile was not associated with malaria infection in a model with HIV infection and site of recruitment. In the final model, the risk of malaria infection was higher among women recruited at Nchelenge health centre (adjusted odds ratio = 1.81; 95 % CI, 1.38–2.37, *P* < 0.001) and

**Table 4:** Predictors of malaria infection diagnosed by PCR among pregnant women of Nchelenge district in Zambia

Potential risk factor category	Number in each category	%	Unadjusted OR	*P* value	Adjusted OR for multivariate analysis	*P* value	Adjusted OR for final model	*P* value
Total	1074	100	1074		1068	<0.001	1072	<0.001
Recruitment site				<0.001		<0.001		<0.001
Kashikishi	737	68.6	1.00		1.00		1.00	
Nchelenge	337	31.4	1.80 (1.37–2.35)		1.89 (1.43–2.50)		1.81 (1.38–2.37)	
Age group				<0.001		0.145		
≤20	295	27.5	1.00		1.00			
21–25	304	28.3	0.51 (0.37–0.72)		0.63 (0.41–0.98)			
26–30	244	22.7	0.51 (0.36–0.73)		0.67 (0.41–1.20)			
≥30	231	21.5	0.45 (0.32–0.64)		0.57 (0.34 –0.95)			
Marital status				0.043		0.412		
Single	201	18.7	1.00		1.00			
Married^a^	873	81.3	0.72 (0.52–0.99)		1.18 (0.78–1.80)			
Gravidity				<0.001		<0.069		
Primigravidae	259	24.1	1.00		1.00			
Secundigravidae	165	15.4	0.74 (0.48–1.11)		0.80 (0.48–1.33)			
Multigravidae	650	60.5	0.50 (0.37–0.68)		0.58 (0.35–0.95)			
Bed-net ownership				0.015		0.640		
No	547	50.9	1.00		1.00			
Yes	527	49.1	0.74 (0.58–0.94)		1.13 (0.72–1.73)			
Bed-net usage (1071)				0.006		0.213		
No	659	61.5	1.00		1.00			
Yes	412	38.5	0.75 (0.48–0.91)		0.74 (0.48–1.18)			
IRS in past 12 months (1025)^b^				0.257				
No	800	78.1	1.00					
Yes	225	21.9	1.19 (0.88–1.61)					
Wealth quintiles (1072)				0.072		0.027*		
Lowest	216	20.1	1.00		1.00			
Second	219	20.4	0.80 (0.54–1.17)		0.73 (0.50–1.09)			
Middle	209	19.5	0.91 (0.61–1.34)		0.76 (0.51–1.15)			
Fourth	214	20.0	1.08 (0.73–1.60)		0.90 (0.59–1.38)			
Highest	214	20.0	0.64 (0.44–0.95)		0.52 (0.35–0.80)			
HIV infection				0.057		0.025		0.045
Negative	936	87.1	1.00		1.00		1.00	
Positive	138	12.9	1.30 (0.91–1.88)		1.62 (1.09–2.39)		1.46 (1.00–2.13)	

Totals of individual variables less than 1074 (available results for malaria diagnosed by polymerase chain reaction) are indicated in the first column. These were due to missing values in individual variables

*IRS* Indoor residual spraying, *HIV* human immunodeficiency virus

^a^The group included women who had been married before (divorced/separated or widowed)

^b^Total number was 1025 due to the exclusion of the ‘unknown’ response. Some women did not know if IRS had been applied to their houses due to occupying them in less than 12 months

* When wealth quintile was put in the model with HIV and site of recruitment it was not significantly associated with malaria infection at *P* < 0.05 and was therefore excluded from the final model. There was no interaction between HIV infection and site of recruitment in the final model (*P* = 0.751)

HIV-infected women across both centres (adjusted odds ratio = 1.46; 95 % CI 1.00–2.13, *P* = 0.045). There was no interaction between HIV status and site of recruitment (*P* = 0.751).

## Discussion

This study provides a robust estimate of the prevalence of peripheral malaria infection among pregnant women of Nchelenge District. A high burden of *P. falciparum* malaria diagnosed by PCR was observed in this population. The prevalence of malaria detected by microscopy was much lower than that detected by PCR, suggesting that a considerable proportion of this study population had sub-microscopic infections (smear negative but PCR positive). Sub-microscopic infections have been associated with placental malaria and decreased maternal haemoglobin [41]. Although no association was found between sub-microscopic infections and adverse maternal and foetal outcomes, the importance of these infections in pregnancy cannot be ignored in malaria policy formulation, especially when factors such as their role in maternal morbidity and malaria transmission are considered [41]. Moreover, pregnant women are a significant reservoir of gametocyte transmission and will require particular attention in elimination efforts [42].

Seven samples that were positive by microscopy in this study were found negative by PCR. Because PCR diagnostic method is more precise than standard microscopy [43], we would expect that a malaria sample found positive by microscopy would also be positive by PCR. The observation above could be attributed to a number of factors including malaria infection due to other *Plasmodium* species and insufficient DNA extraction. A negative PCR test result may be an outcome if the amount of blood collected on filter paper was less than 25 µl, coupled with low parasitaemia.

The prevalence of malaria infection by microscopy was very similar to the prevalence of parasitaemia observed in children less than five-years-old in 2012 (32.1 %, 114/356) in the MIS in Luapula Province [20]. Another study conducted in the same area among children less than 10 years of age reported a similar parasite prevalence, 30.2 % (236/782) by microscopy [29].

Analysis of monthly-reported district-level malaria cases among pregnant women showed that MiP in Zambia decreased between 2010 and 2013, although persistent hot spots were reported in the southeast and northeast of the country [44].

The prevalence of malaria among 375 pregnant women in the study conducted in Nchelenge District between February and April 2013 by Siame et al. was much lower than what was observed in the current study, 15.0 % by microscopy and 22.0 % by PCR [45]. The differences in the prevalence of malaria in the two studies could be attributed to a number of factors. Firstly, in the current study women attending the Nchelenge and Kashikishi health centres were recruited, whereas in the study by Siame et al. a third health centre, Kabuta, was included in addition to these two. Secondly, in the study by Siame et al. pregnant women who sought ANC services were included, regardless of visit number; in this study, only first ANC attendees who had not taken anti-malarial treatment in the previous month before recruitment were enrolled. Some of the participants who were included in the study by Siame et al. had received IPTp-SP during their earlier ANC visit(s) as well as anti-malarial treatment before enrolment, and it is unclear what time period may have elapsed between treatment and subsequent screening. The prevalence of malaria is likely lower in a group of women exposed to any IPTp-SP because even one dose of IPTp-SP has been shown to protect against maternal parasitaemia [46].

There was a difference in malaria infection and parasite densities among HIV-infected and HIV-uninfected women, a finding similar to other studies [23, 24, 47–51]. The proportion of malaria infection detected by microscopy and by PCR in this study was consistently higher in HIV-infected than HIV-uninfected women. HIV-infected women had a 46 % increased risk of malaria infection detected by PCR at first ANC visit (*P* = 0.045). However, the association between HIV and malaria infection was not statistically significant.

Previous studies have reported stronger association between HIV and peripheral malaria detected by microscopy in pregnant women. A hospital-based study in Zimbabwe in 2000–2001 reported a high odds of malaria parasitaemia in HIV-infected women compared to HIV-uninfected women (OR 3.96; 95 % CI, 2.42–6.46) [50]; a study in Rwanda conducted between 1992 and 1993 reported that the risk of parasitaemia was moderately higher in HIV-infected compared to HIV-uninfected women (adjusted risk ratio 1.40; 95 % CI, 1.1–1.6, *P* = 0.016) [47]; a similar finding was reported from Kenya in a study conducted between 1996 and 1999 among HIV-infected women in the third trimester (risk ratio 1.70; 95 % CI, 1.52–1.90) and at delivery (risk ratio 1.56; 95 % CI, 1.34–1.81) [51]. The prevalence of malaria parasitaemia among HIV-infected women at first ANC visit was higher than among HIV-uninfected women in a study in Malawi conducted between 1987 and 1990 (56.6 versus 43.6 %, *P* < 0.001) [49].

The use of antiretroviral therapy was not stated in all the studies above and, considering the years in which these studies were conducted, it can be assumed that coverage of antiretroviral therapy among pregnant women was very low if not non-existent. In the current study, however, the coverage of antiretroviral therapy at first ANC visit was 42.7 % among all HIV-positive women, newly-tested and known HIV-positive cases. Some of the women who were screened in the current study were receiving co-trimoxazole prophylaxis although coverage data of cotrimoxazole at recruitment was not collected. However, data from delivery showed that 37 % of HIV-infected study participants had received cotrimoxazole prophylaxis during pregnancy. Thus, the relatively a weaker association between HIV-infected status and malaria parasitaemia observed in the current study compared to the previously reported studies could be due to the fact that some of the HIV-infected women were on ARV and/or cotrimoxazole prophylaxis.

There was no difference in the geometric mean of parasite density in HIV-infected women on antiretroviral therapy and women who were not on treatment. This may be attributable to newer HIV infections. Women who were not already on antiretroviral therapy may not have progressed to a state of highly compromised immunity since some of them tested positive for the first time at their ANC booking. Furthermore, eligibility to receive antiretroviral drugs in this community was linked to CD4 count. Therefore, if women known to be HIV-positive were not on therapy, it was likely that their CD4 count was above 500 cells per cubic millimetre of blood.

The fact that bed net usage was much lower in HIV-infected women than in HIV-uninfected women, despite there being no significant difference in bed net ownership in the two groups, is surprising because one might expect the opposite. It is unclear if low bed net usage confers an additional risk of malaria infection in HIV-infected women in this study group as would be expected considering that no association was found between bed net use and malaria infection in this study. The significant difference in use of bed nets suggests that there could be differences in disease prevention behaviours between the two groups.

In the univariate analysis, the risk of infection was strongly associated with gravidity and age, but this was not observed in the multivariate analysis. Studies have shown that in malaria-endemic areas, the prevalence of malaria, both clinical and asymptomatic, is highest in young women and primigravidae and paucigravidae (primigravidae and secundigravidae combined) [2, 52, 53]. This is due to acquisition of semi-immunity that is gravidity-dependent such that malaria infection tends to be less prevalent and less severe among multigravidae [2, 6, 52]. However, HIV infection has been shown to impair the ability of multigravidae to manage malaria infection [23, 51]. The fact that HIV prevalence was highest among multigravidae in this study population is a plausible explanation for the fact that there was no association between parity and malaria infection in multivariate analysis.

The low coverage of IRS and bed net use in an area of intense transmission may partially explain the high prevalence of malaria infection. Despite the progress that has been made in scaling-up malaria control interventions, a high burden of malaria still remains in Nchelenge District [19, 54]. Several reasons may underlie this observation including population movements from high endemic areas (internally and across borders), increasing parasite resistance to insecticides [29, 54], as well as homes having been built close to water sources that serve as breeding grounds for *Anopheles* mosquitoes.

The risk of malaria infection was higher among women at Nchelenge health centre compared to Kashikishi health centre. Since there was no difference in socio-demographic characteristics and HIV infection at the two sites, this difference in the risk of malaria infection may be attributable to the intensity of malaria transmission within the catchment areas of Kashikishi and Nchelenge health centres. Apart from providing health services to the people living close to the Lake Mweru, Nchelenge health centre also provides health services to people in villages that are close to a rubber plantation, the Kenani River, and other in-land water bodies in the Robert Mutepuka village area which provide more breeding sites for mosquitoes.

Malaria transmission intensity is known to decrease during the winter months, April to August, in Nchelenge District [18]. Thus, the estimated prevalence of MiP from this study conducted during the hot rainy season from November to April is probably higher than the prevalence at other times of the year. Zambia has different malaria transmission zones ranging from very low, to low-moderate and moderate-high [55]. Thus, the prevalence of malaria estimated in this study can only be cautiously extended to areas with similar transmission patterns.

The prevalence of *P. falciparum* malaria detected by PCR in pregnant women of Nchelenge District is high. HIV infection increased the risk of malaria infection, although our sample size was not large enough to conclude this risk was statistically significant. Robust vector control, the provision of IPTp-SP, and community education on the importance of using the available interventions to prevent and to treat MiP are vital to reducing the malaria burden in pregnancy. The high prevalence of malaria infection that we observed in this population suggests that past prevention efforts have had limited impact in pregnant women.

## Additional file

**Additional file 1.** STROBE Statement.

## Abbreviations

ANC: antenatal care
CIs: confidence intervals
DNA: deoxyribonucleic acid
IPTp: intermittent preventive treatment in pregnancy
MiP: malaria in pregnancy
MIS: malaria indicator survey
PCR: polymerase chain reaction
SP: sulfadoxine-pyrimethamine
STI/RTI: sexually transmitted and reproductive tract infections
WBCs: white blood cells
